# *Salmonella enterica *serotype Virchow associated with human infections in Switzerland: 2004-2009

**DOI:** 10.1186/1471-2334-11-49

**Published:** 2011-02-23

**Authors:** Mario Bonalli, Roger Stephan, Ursula Käppeli, Nicole Cernela, Luzius Adank, Herbert Hächler

**Affiliations:** 1 Institute for Food Safety and Hygiene, University of Zurich, Winterthurerstr. 272, 8057 Zurich, Switzerland

## Abstract

**Background:**

Salmonellosis is one of the most important foodborne diseases and a major threat to public health. *Salmonella *serotype Virchow ranks among the top five serovars in Europe.

**Method:**

A total of 153 strains isolated from different patients from 2004 through 2009 in Switzerland were further characterized by (i) assessing phenotypic antibiotic resistance profiles using the disk diffusion method and (ii) by genotyping using pulsed-field gel electrophoresis (PFGE) after macrorestriction with *Xba*I in order to evaluate strain relationship.

**Results:**

The relative frequency of *S*. Virchow among other *Salmonella *serovars varied between 4^th ^to 8^th ^rank. The annual incidence ranged from 0.45/100'000 in 2004 to 0.40/100'000 in 2009. A total of 48 strains (32%) were resistant to one to 3 antimicrobials, 54 strains (36%) displayed resistance patterns to more than three antibiotics. No trend was identifiable over the years 2004 to 2009. We found a high prevalence (62%) of nalidixic acid resistant strains, suggesting an equally high rate of decreased fluoroqionolone susceptibility, whereas intermediate resistance to ciprofloxacin was negligible. Two strains were extended spectrum β-lactamase (ESBL) producers. Analysis of PFGE patterns uncovered a predominant cluster (similarity coefficient above 80%) consisting of 104 of the 153 strains.

**Conclusion:**

The worldwide increase of antibiotic resistances in *Salmonella *is an emerging public health problem. For Switzerland, no clear trend is identifiable over the years 2004 to 2009 for *S*. Virchow. Antimicrobial susceptibility and resistance profiles varied considerably within this period. Nevertheless, the situation in Switzerland coincided with findings in other European countries. Genotyping results of this strain collection revealed no evidence for an undetected outbreak within this time period.

## Background

Non-typhoidal salmonellae are worldwide responsible for numerous foodborne infections and are a major threat to public health [1,2]. Based on the lipopolysaccharide (O antigen) and the flagellar structures (H antigen) S*almonella *spp. are divided into greater than 2500 serotypes, of which only few obtain substantial relevance to humans [3]. In Europe, as well as in Switzerland, *Salmonella *serotype Virchow ranks among the five most frequent serovars and has been reported to be poultry associated [4-9]. As with other enteritic salmonellae, infections due to *S*. Virchow commonly manifest themselves as self-limiting gastroenteritis, but severe invasive infections can also occur which then need antibiotic treatment [10]. Whereas fluoroquinolones are used to treat extraintestinal infections in adults, third generation cephalosporins are drugs of choice in children. Alarmingly, in the last decade numerous European countries reported emergence of *S*. Virchow strains resistant to the above mentioned antimicrobials, leading to treatment failures [9,11,12]. Resistance to quinolones is either mediated by chromosomal mutations in genes encoding DNA gyrase or topoisomerase IV or in genes affecting the uptake or efflux of drugs [13]. Plasmid associated quinolone corrupting genes such as *aac(6')-Ib-cr or qnr *etc., are also known to cause reduced susceptibility. Resistance to third generation cephalosporins is mediated by extended spectrum β-lactamases (ESBL), which are plasmid encoded or integron associated [14].

In this study, a total of 153 *S*. Virchow strains isolated from different patients from 2004 through 2009 in Switzerland were further characterized by (i) assessing phenotypic antibiotic resistance profiles using the disk diffusion method and (ii) by genotyping using pulsed-field gel electrophoresis (PFGE) after macrorestriction with *Xba*I in order to evaluate strain relationship.

## Methods

### Strains

Human salmonellosis is a reportable disease in Switzerland and isolates have to be submitted to the National Centre for Enteropathogenic Bacteria (NENT). The NENT - in cooperation with the Swiss Federal Office of Public Health - collects clinical data and carries out final serological identification by slide agglutination with commercial antisera according to the Kauffmann-White scheme. Over the years 2004 through 2009, 10395 *Salmonella *strains were submitted to the NENT. From this collection of strains 153 *S*. Virchow strains (1,5%) from different patients (multiple isolates from same patient excluded, but family members not excluded) were selected and integrated in this study. All samples had been collected by hospitals or family doctors.

### Antimicrobial susceptibility testing

The strains were tested for antimicrobial resistance by the disk diffusion method according to the Clinical and Laboratory Standards Institute (CLSI) [15]. The panel of antibiotic disks (Becton, Dickinson and Company, Maryland, USA) consisted of ampicillin (AM10), amoxicillin/clavulanic acid (AMC30), cephalothin (CF30), cefotaxime (CTX30), ciprofloxacin (CIP5), gentamicin (GM10), tetracycline (Te30), streptomycin (S10), chloramphenicol (C30), kanamycin (K30), nalidixic acid (NA30), sulfamethoxazole (SMZ), and trimethoprim (TMP5). The strains were classified as resistant, intermediate or susceptible to each antibiotic agent according to the CLSI criteria [15].

For presumptive ESBL producers - strains that produced synergistic inhibition zone enlargements between adjacent discs containing an oxyimino cephalosporin and clavulanic acid, respectively - Etest ESBL (bioMérieux, Marcy l'Etoile, France) tests, containing cefotaxime (CT/CTL), ceftazidime (TZ/TZL), or cefepime (PM/PML) alone and in combination with clavulanic acid, were performed for confirmation according the manufacturer's guidelines.

### Genotyping

Pulsed-field gel electrophoresis (PFGE) was performed by following the CDC PulseNet protocol http://www.cdc.gov/pulsenet/protocols.htm with minor modifications. In brief, strains were grown on blood agar at 37°C over night. Colonies from blood agar were resuspended in cell suspension buffer (OD600 = 1). The bacterial cell suspension was mixed with 400 μl of 1.4% Pulsed Field Certified Agarose (BIO-RAD, Munich, Germany) and cells were lysed by proteinase K treatment over night. After lysis the plugs were washed twice for 30 min in ultrapure water and 4 times for an hour in Tris-EDTA (TE) buffer. After washing with TE buffer, DNA agarose plugs were incubated over night in the presence of *Xba*I (Roche, Mannheim, Germany) following the manufacturer's instructions. Restricted DNA in plug slices was separated in a 1% SeaKem Gold (BioConcept, Allschwil, Switzerland) agarose gel at 6 V/cm in 0.5 M Tris-Borate-EDTA buffer cooled to 12°C in a CHEF-DR III system (BIO-RAD, Munich, Germany). The pulse times were ramped from 2 to 64 sec for 20 h at an angle of 120°. Gels were stained with ethidium bromide and visualized under UV light transillumination with Gel Doc (BIO-RAD, Munich, Germany) and analysed with BioNumerics software (Applied Maths, Sint-Martens-Latem, Belgium). Pair wise similarities between the *Xba*I pulsed-field patterns were calculated by the DICE's similarity coefficient. Clustering was based on the unweighted pair-group method with averages (UPGMA), setting tolerance and optimization each at 1.5%. We used *Salmonella *Braenderup strain H9812 (ATCC BAA 664) as a reference strain.

## Results

### Clinical and epidemiological data

The 153 *S*. Virchow strains were isolated from 124 faecal samples (81%), 17 blood samples (11.1%), two urine samples (1.3%), two swabs (1.3%), and one synovial puncture (0.7%). For 7 strains (4.6%) the origin was unknown. The relative frequency of *S*. Virchow among other *Salmonella *serovars varied between 4^th ^to 8^th ^rank. The annual incidence ranged from 0.45/100'000 in 2004 to 0.40/100'000 in 2009 (lowest incidence: 0.39/100'000 in 2006, highest incidence: 0.47/100'000 in 2007).

The age of the patients was known for 99% of the isolates submitted. Fourteen percent of the *S*. Virchow strains were submitted from patients aged 5 years and younger (incidence: 0.9/100'000), 3.5% from patients aged 6 to 14 years (incidence: 0.13/100'000), 74.5% from patients aged 15 to 65 years (incidence: 0.4/100'000) and 7% from patients older than 65 years (incidence: 0.17/100'000). The incidence among infants of 5 years and younger was seven fold higher than that among children aged 6 to 14 years. The incidence of people between 15 and 65 years was three fold higher than that of children aged 6 to 14 years. Of the patients infected with *S*. Virchow 52% were female and 48% were male.

Of all patients, 35 (23%) had travelled abroad, the travelling status was unknown for 117 patients (76%), and only one person confirmed not having visited a foreign country within two weeks prior to infection. Eleven persons (7.2%) had visited Egypt, 7 (4.6%) Thailand, 3 (2%) India, 2 (1.3%) Kenya, 2 (1.3%) Asia. The following countries had been visited by one person each (0.7%): Guinea, Jordan, Senegal/Gambia, China, Sri Lanka and Tanzania. Four patients (2.6%) affirmed having visited a foreign country within two weeks prior to infection start, but gave no further information about the destination. As many as 82.5% who had travelled to a foreign country within two weeks before disease outbreak, were between 15 and 65 years old, 7.5% were younger than 6 years, another 7.5% were aged from 6 to 14 years, and 2.5% were older than 65. Subtracting the number of patients with travel background from the total of the patients lowered the average incidence from 0.42/100'000 to 0.34/100'000. Turning the attention on the 6 to 14 year old children, the incidence reduced even from 0.13/100'000 to 0.07/100'000 by subtracting those who stayed abroad. Regarding the 15 to 65 year old patients, the incidence showed similar behaviour and decreased from 0.41/100'000 to 0.3/100'000 while the incidence of other age-groups exhibited only minor changes.

### Antimicrobial susceptibility

**Figure 1:**
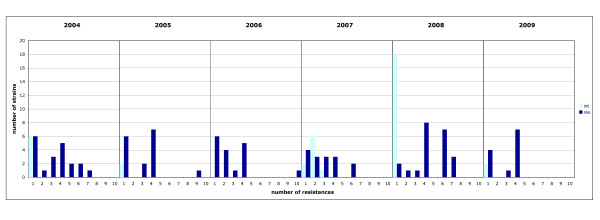
**Assigned to the number of antibiotic resistances (1 to 10), for each year, the number of resistant strains are constituted as dark blue, intermediate strains as light blue columns, respectively**.

A high prevalence of resistant (resistant to 1 to 3 antimicrobials) and multi drug resistant (MDR = resistant to more than 3 antibiotics) *S*. Virchow strains was detected. For calculation of percentages, intermediate susceptibility was counted as "susceptible". A total of 48 strains (32%) were resistant and 54 strains (36%) displayed MDR, whereas only 50 strains (33%) showed full susceptibility. There is no clear trend identifiable over the years 2004 to 2009. Antimicrobial susceptibility and resistance profiles varied considerably (Figure 1). Thus in 2008, 67% of all isolates exhibited MDR, and only 19% were susceptible to all antibiotic agents tested. However, in 2009 only 25% of all strains displayed MDR but 57% showed no resistances.

**Figure 2:**
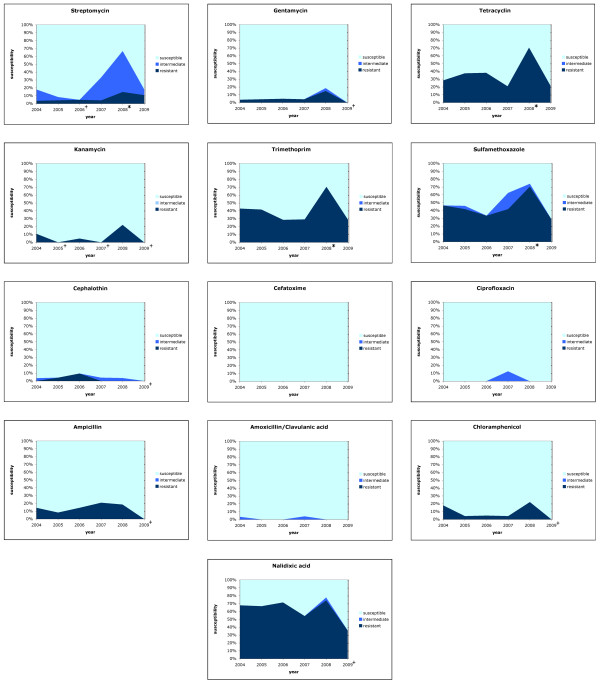
**Resistance of *S*. Virchow strains isolated over the years 2004 trough 2009 in Switzerland**. Each box shows the percentage fraction of the resistant, intermediate and susceptible sub-population over time for one antimicrobial. Values significantly (p < 0.05) deviating from average are marked with * (more resistant) or + (less resistant), respectively.

We found 62% of all *S*. Virchow strains resistant to nalidixic acid, while 98% were susceptible to ciprofloxacin, and only 2% (3 isolates) were intermediate according to CLSI criteria [15]. As many as 49% of the isolates were resistant to sulfamethoxazole, 41% to trimethoprim and 36% to tetracycline. All strains were susceptible to cefotaxime, and only 1.3% were resistant to amoxicillin/clavulanic acid. During the years 2004 to 2007 the resistance situation remained consistent. In 2008, for several antimicrobials the percentage of resistant strains was significantly higher (p < 0.05) than in the preceding years. Interestingly, multiple percentages during 2009 lay beneath the average values of the years 2004 to 2009 (p < 0.05) (Figure 2). Hence, there were no significant trends in resistance development observable for any antibiotic.

Two *S*. Virchow strains (05N2379 and 06N1956) produced ESBL. Both strains tested positive with the confirmatory Etest ESBL (ratio CT/CTL = 62,5; ratio TZ/TZL >340; ratio PM/PML >6).

### Genetic relationship among Swiss *S*. Virchow isolates

**Figure 3:**
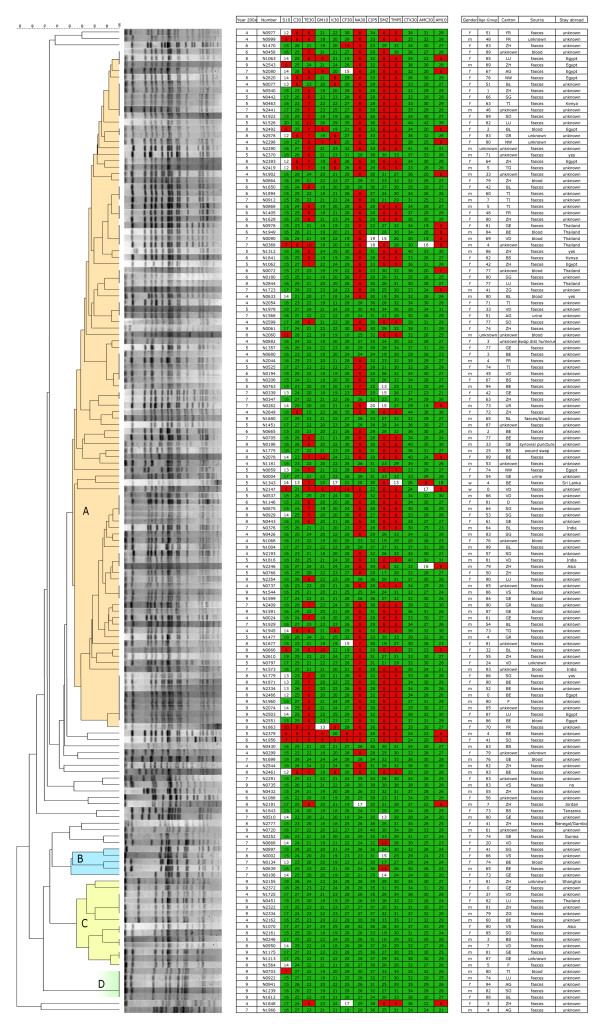
**Anamnestic data, resistance profiles and PFGE patterns of the 153 *S*. Virchow strains**. PFGE clusters are faintly coloured and marked A-D. Strains resistant, intermediate or susceptible to a specific antibiotic are highlighted in red, white, or green, respectively.

PFGE analysis of the 153 *Xba*I-digested strains revealed 104 pulsotypes with 14 to 22 DNA fragments ranging from 20,5 kb to 1135 kb. Five and more related strains defined the formation of a cluster. We discovered four pattern clusters (A-D, Figure 3), when strains were considered related if their similarity coefficient exceeded 80%. There were no correlations regarding gender, year of isolation, age-group or stay-abroad, and no other common properties were found for isolates within a certain cluster. A number of pulsotypes incorporated several indistinguishable isolates. Their content ranged from 2 to 8 indistinguishable isolates (Figure 3).

## Discussion

During the study period, 1.5% of all *Salmonella *strains submitted to the NENT belonged to the serotype Virchow. This amounted to a prevalence of 0.45 (2004) to 0.40 (2009) cases per 100'000 population, and was within the average in Europe [4,5]. For a comparison, the prevalence of total salmonellosis in Switzerland was 26 (2004) to 25 (2009) cases per 100'000 population. Whereas in France the annual number as well as the corresponding rank decreased during 2005 to 2008 [5], in Switzerland the number of cases associated with *S*. Virchow infections and its rank remained constant from 2004 through 2009.

Conspicuously, the incidence of infected children aged 0 to 5 years was seven fold higher than that in children older than 5 years. Reasons may be an insufficient immune defence that lowers the minimal infective dose, as the immune system has not yet fully developed. Regarding the age-group from 15 to 65, we discovered a three fold higher incidence compared to the incidence of persons aged older than 65. This could be linked to considerable differences in travelling activity. Thus, 25% of all patients between 15 and 65 years of age had been visiting a foreign country within 2 weeks before infection. Even 50% of the infections among 6 to 14 years old children were associated with travelling, whereas other age-groups could hardly be linked to a stay-abroad. Upon the present investigation, no case control study could be performed, and, unfortunately, exact data about travel destinations were available only with 31 of the 153 cases. Nevertheless, 16 and 15 out of the 31 cases could be traced back to African and Asian countries, respectively.

In this study, *S*. Virchow strains isolated from blood samples, and presumably associated with invasive manifestation, were enclosed. Interestingly, we did not receive any blood samples derived from children up to 5 years. In contrast, 16% blood samples were submitted from adult patients of the 15 to 65 age group. Similar findings were published in 2005 from a university hospital in Crete (Greece). Galanakis and his team found 41% of all patients infected with non-thyphoidal salmonellae aged under 15 years, but only 4.06% suffered from an invasive disease. Adult patients (> 15 years) suffered twice as much from an invasive manifestation [16]. However, Parry reported about sub-Saharan Africa, where invasive infections caused by enteritic serotypes are particularly common in children under 5 years [17]. Reasons for such divergent findings are unknown, but differences between developed and developing countries seem to exist.

Genotyping revealed 104 strains with a high degree (> 82%) of relationship, summarised in cluster A, which constituted the major part of all isolates. Additionally, several groups of samples were discovered, representing indistinguishable pulsotypes. Strains belonging to cluster A were collected within the years 2004 to 2009 and originated from different countries. This indicates, that these closely related isolates are highly established and widespread.

Indistinguishable patterns give evidence to small outbreaks or point sources, that spread a certain strain over several years. This becomes particularly apparent regarding non-discriminable isolates derived from patients that stayed in the same foreign country. As already mentioned, most of the strains from patients with a travelling background had returned from the North-African or Asian region. Close clonal relationship between strains from those regions is exemplified by two PFGE sub-clusters within the large Cluster A. One sub-cluster is associated with Egypt (Figure 3, strains no. 8-N1063, 9-N2543, 7-N2080, 8-N2820, 8-N2492, 8-N2393), the other with Thailand (Figure 3, strains no. 6-N0978, 6-N1949, 7-N0090, 7-N0368).

The number of resistant (31.5%) and multiresistant (35.5%) strains is considerable. Similar rates from European countries have already been published. In 2004 a study performed by Meakins and co-authors revealed 73% of all *S*. Virchow strains resistant to at least one antimicrobial agent [18]. Threlfall, in a European multi-centre study with over 27000 cases of salmonellosis in 2000, discovered 36% multi drug resistant isolates [4]. Strikingly, in our study almost all MDR isolates lay within cluster A (Figure 3). Similarities in the resistance situation between the European Union and Switzerland indicate a close relationship of strains belonging to cluster A and strains responsible for the high level of resistance in Europe.

A high prevalence of nalidixic acid resistant strains was found within cluster A, whereas nearly no resistance to nalidixic acid could be observed among the remaining isolates. A similar behaviour for resistances to tetracycline, sulfonamide and trimetoprim was noted.

Fluoroquinolones and third-generation cephalosporins are drugs-of-choice for invasive *Salmonella *infections. During the past years numerous studies reported the emergence of fluoroquinolone or third-generation cephalosporine resistant strains [9,11,12]. We found a high prevalence of isolates resistant to nalidixic acid but no full resistance against ciprofloxacin, and only 2% of isolates being intermediate over the whole study period. Because of the intersecting resistance mechanisms against quinolones and fluoroquinolones, the behaviour against ciprofloxacin must be discussed in association with that to nalidixic acid [19]. Because of repeated reports of fluoroquinolone treatment failure following disease caused by *Salmonella *strains that were susceptible to fluoroquinolones using CLSI criteria, but were simultaneously resistant to nalidixic acid, several authors called for re-evaluation of the CLSI breakpoints for fluoroquinolones involving salmonellae [20-22]. Consequently, comments no. 2, 18, and 20 were added to Table Two A in CLSI documents, as can be seen, for instance, in the 2008 edition [15]. They serve as a warning, that fluoroquinolone-susceptible but nalidixic acid-resistant extraintestinal salmonellae must be considered reduced-susceptible to fluoroquinolones. By the comments, the reader is also urged to perform a nalidixic acid susceptibility test, in order to become aware of such strains. Based on these arguments, the 62% naldixic acid-resistant *S*. Virchow isolates of the present study have to be considered reduced-susceptible to ciprofloxacin, and thus prone to cause a significant rate of treatment failure. The observation of a low rate of "obvious" resistance to fluoroquinolones is in accordance with data from other European countries [4,5]. As we discovered only two ESBL producing isolates in the years 2005 and 2006, the occurrence of such strains in Switzerland are rather rare events compared to other European countries [5,11,23].

In many countries, *S*. Virchow infections are poultry associated [6-9]. In Switzerland, however, there is a favourable situation in poultry flocks with respect to *Salmonella *in general and *S*. Virchow in particular [24]. However, 52% of all poultry meat is imported and 71% of the imports originate from Brazil, Germany and France. From all these countries, detection of *S*. Virchow in poultry products has been reported [7,25,26]. On this basis, one might speculate, that a majority of the *S*. Virchow infections in Switzerland may be linked to travelling activities and/or consumption of imported poultry meat. This, however, would have to be further investigated by additional case control- and epidemiological studies.

## Conclusion

The worldwide increase of antibiotic resistances in *Salmonella *is an emerging public health problem. For Switzerland, no clear trend is identifiable over the years 2004 to 2009 for *S*. Virchow. Antimicrobial susceptibility and resistance profiles varied considerably within this period. Nevertheless, the situation in Switzerland coincided with findings in other European countries. Genotyping results of this strain collection revealed no evidence for an undetected outbreak within this time period

## Competing interests

The authors declare that they have no competing interests.

## Authors' contributions

RS, LA and HH designed the study. MB, NC, UK have done the phenotypic and genotypic characterization of the strains. MB and RS drafted the manuscript. All authors read, commented on and approved of the final manuscript.

## Pre-publication history

The pre-publication history for this paper can be accessed here:

http://www.biomedcentral.com/1471-2334/11/49/prepub

## References

[B1] Rabsch W Tschape H Baumler AJ Non-typhoidal salmonellosis: emerging problems Microbes Infect 2001 3 237 247 10.1016/S1286-4579(01)01375-2 11358718

[B2] Olsen SJ Bishop R Brenner FW Roels TH Bean N Tauxe RV Slutsker L The changing epidemiology of salmonella: trends in serotypes isolated from humans in the United States, 1987-1997 J Infect Dis 2001 183 753 761 10.1086/318832 11181152

[B3] Weinberger M Solnik-Isaac H Shachar D Reisfeld A Valinsky L Andorn N Agmon V Yishai R Bassal R Fraser A Salmonella enterica serotype Virchow: epidemiology, resistance patterns and molecular characterisation of an invasive Salmonella serotype in Israel Clin Microbiol Infect 2006 12 999 1005 10.1111/j.1469-0691.2006.01466.x 16961637

[B4] Threlfall EJ Fisher IS Berghold C Gerner-Smidt P Tschape H Cormican M Luzzi I Schnieder F Wannet W Machado J Edwards G Antimicrobial drug resistance in isolates of Salmonella enterica from cases of salmonellosis in humans in Europe in 2000: results of international multi-centre surveillance Euro Surveill 2003 8 41 45 12631974 10.2807/esm.08.02.00400-en

[B5] Weill FX Le Hello S Rapport d'activité annuel 2008 Centre National de Référence des Salmonella 2008

[B6] Riano I Moreno MA Teshager T Saenz Y Dominguez L Torres C Detection and characterization of extended-spectrum beta-lactamases in Salmonella enterica strains of healthy food animals in Spain J Antimicrob Chemother 2006 58 844 847 10.1093/jac/dkl337 16935865

[B7] Weill FX Lailler R Praud K Kerouanton A Fabre L Brisabois A Grimont PA Cloeckaert A Emergence of extended-spectrum-beta-lactamase (CTX-M-9)-producing multiresistant strains of Salmonella enterica serotype Virchow in poultry and humans in France J Clin Microbiol 2004 42 5767 5773 10.1128/JCM.42.12.5767-5773.2004 15583311 PMC535271

[B8] Adak B Threlfall EJ Outbreak of drug-resistant *Salmonella *Virchow phage type 8 infection CDR Weekly 2005 15 9 2 3

[B9] Bertrand S Weill FX Cloeckaert A Vrints M Mairiaux E Praud K Dierick K Wildemauve C Godard C Butaye P Clonal emergence of extended-spectrum beta-lactamase (CTX-M-2)-producing Salmonella enterica serovar Virchow isolates with reduced susceptibilities to ciprofloxacin among poultry and humans in Belgium and France (2000 to 2003) J Clin Microbiol 2006 44 2897 2903 10.1128/JCM.02549-05 16891509 PMC1594617

[B10] Mani V Brennand J Mandal BK Invasive illness with Salmonella virchow infection Br Med J 1974 2 143 144 10.1136/bmj.2.5911.143 4856881 PMC1610336

[B11] Herrera-Leon S Gonzalez-Sanz R Rodriguez I Rodicio MR Echeita MA Spread of a multiresistant CTX-M-9-producing Salmonella enterica serotype Virchow phage type 19 in Spain Eur J Clin Microbiol Infect Dis 2010 29 901 905 10.1007/s10096-010-0939-6 20446012

[B12] Yates C Amyes S Extended-spectrum beta-lactamases in non-typhoidal Salmonella spp. isolated in the UK are now a reality: why the late arrival? J Antimicrob Chemother 2005 56 262 264 10.1093/jac/dki237 16000403

[B13] Solnik-Isaac H Weinberger M Tabak M Ben-David A Shachar D Yaron S Quinolone resistance of Salmonella enterica serovar Virchow isolates from humans and poultry in Israel: evidence for clonal expansion J Clin Microbiol 2007 45 2575 2579 10.1128/JCM.00062-07 17596371 PMC1951243

[B14] Dierikx C van Essen-Zandbergen A Veldman K Smith H Mevius D Increased detection of extended-spectrum beta-lactamase producing *Salmonella enterica *and *Escherichia coli *isolates from poultry Vet Microbiol 2010 145 273 278 10.1016/j.vetmic.2010.03.019 20395076

[B15] Clinical and Laboratory Standards Institute Performance Standards for Antimicrobial Susceptibility Testing; Eighteenth Informational Supplement CLSI document M100-S18 2008, Wayne, PA

[B16] Galanakis E Bitsori M Maraki S Giannakopoulou C Samonis G Tselentis Y Invasive non-typhoidal salmonellosis in immunocompetent infants and children Int J Infect Dis 2007 11 36 39 10.1016/j.ijid.2005.09.004 16564718

[B17] Parry CM Antimicrobial drug resistance in Salmonella enterica Curr Opin Infect Dis 2003 16 467 472 10.1097/00001432-200310000-00014 14502000

[B18] Meakins S Fisher IS Berghold C Gerner-Smidt P Tschape H Cormican M Luzzi I Schneider F Wannett W Coia J Antimicrobial drug resistance in human nontyphoidal Salmonella isolates in Europe 2000-2004: a report from the Enter-net International Surveillance Network Microb Drug Resist 2008 14 31 35 10.1089/mdr.2008.0777 18366323

[B19] Cloeckaert A Chaslus-Dancla E Mechanisms of quinolone resistance in Salmonella Vet Res 2001 32 291 300 10.1051/vetres:2001105 11432420

[B20] Hakanen A Kotilainen P Jalava J Siitonen A Huovinen P Detection of decreased fluoroquinolone susceptibility in salmonellas and validation of nalidixic acid screening test J Clin Microbiol 1999 37 3572 3577 10523554 10.1128/jcm.37.11.3572-3577.1999 PMC85694

[B21] Aarestrup FM Wiuff C Molbak K Threlfall EJ Is it time to change fluoroquinolone breakpoints for *Salmonella *spp.? Antimicrob Agents Chemother 2003 47 827 829 10.1128/AAC.47.2.827-829.2003 12543704 PMC151776

[B22] Crump JA Barrett TJ Nelson JT Angulo FJ Reevaluating fluoroquinolone breakpoints for *Salmonella enterica *Typhi and for non-Typhi salmonellae Clin Infect Dis 2003 37 75 81 10.1086/375602 12830411

[B23] Riano I Garcia-Campello M Saenz Y Alvarez P Vinue L Lantero M Moreno MA Zarazaga M Torres C Occurrence of extended-spectrum beta-lactamase-producing Salmonella enterica in northern Spain with evidence of CTX-M-9 clonal spread among animals and humans Clin Microbiol Infect 2009 15 292 295 10.1111/j.1469-0691.2008.02673.x 19175621

[B24] Anonymous Schweizer Zoonosebericht 2008 Bundesamt für Veterinärwesen 2008

[B25] Hernandez T Sierra A Rodriguez-Alvarez C Torres A Arevalo MP Calvo M Arias A Salmonella enterica serotypes isolated from imported frozen chicken meat in the Canary islands J Food Prot 2005 68 2702 2706 16355846 10.4315/0362-028x-68.12.2702

[B26] Atanassova V Matthes S Muhlbauer E Helmuth R Schroeter A Ellendorff F Plasmid profiles of different Salmonella serovars from poultry flocks in Germany Berl Munch Tierarztl Wochenschr 1993 106 404 407 8129697

